# Effect of acute resistance exercise on carotid artery stiffness and cerebral blood flow pulsatility

**DOI:** 10.3389/fphys.2014.00101

**Published:** 2014-03-19

**Authors:** Wesley K. Lefferts, Jacqueline A. Augustine, Kevin S. Heffernan

**Affiliations:** Department of Exercise Science, Syracuse UniversitySyracuse, NY, USA

**Keywords:** arterial stiffness, blood pressure, exercise, wave reflection

## Abstract

Arterial stiffness is associated with cerebral flow pulsatility. Arterial stiffness increases following acute resistance exercise (RE). Whether this acute RE-induced vascular stiffening affects cerebral pulsatility remains unknown. Purpose: To investigate the effects of acute RE on common carotid artery (CCA) stiffness and cerebral blood flow velocity (CBFv) pulsatility. Methods: Eighteen healthy men (22 ± 1 yr; 23.7 ± 0.5 kg·m^−2^) underwent acute RE (5 sets, 5-RM bench press, 5 sets 10-RM bicep curls with 90 s rest intervals) or a time control condition (seated rest) in a randomized order. CCA stiffness (β-stiffness, Elastic Modulus (Ep)) and hemodynamics (pulsatility index, forward wave intensity, and reflected wave intensity) were assessed using a combination of Doppler ultrasound, wave intensity analysis and applanation tonometry at baseline and 3 times post-RE. CBFv pulsatility index was measured with transcranial Doppler at the middle cerebral artery (MCA). Results: CCA β-stiffness, Ep and CCA pulse pressure significantly increased post-RE and remained elevated throughout post-testing (*p* < 0.05). No changes in MCA or CCA pulsatility index were observed (*p* > 0.05). There were significant increases in forward wave intensity post-RE (*p* < 0.05) but not reflected wave intensity (*p* > 0.05). Conclusion: Although acute RE increases CCA stiffness and pressure pulsatility, it does not affect CCA or MCA flow pulsatility. Increases in pressure pulsatility may be due to increased forward wave intensity and not pressure from wave reflections.

## Introduction

Increases in arterial stiffness with age and/or disease increases risk for cardiovascular events such as myocardial infarction and stroke (Sutton-Tyrrell et al., 2005; Mitchell et al., 2010). Increased arterial stiffness also contributes to target organ damage such as renal dysfunction and retinal damage (Katsi et al., 2012; Safar et al., 2012). The elastic properties of the large central arteries (i.e., aorta and carotid) function to dampen the amplitude of fluctuations in pressure and flow, thereby preventing transmission of excess energy into target organs (Mitchell et al., 2011). Similar to the kidney and eye, the brain is a high flow organ particularly susciptible to hemodynamic pulsatility (Mitchell, 2008). Repeated exposure of the cerebral vasculature to pulsatile pressure/flow may precipitate microvascular hypoperfusion and subsequent ischemia contributing to rarefaction, white matter hyperintensities and ultimately cerebrovascular impairment (Mitchell et al., 2005). Recent studies note a strong assocation between arterial stiffness, pressure/flow pulsatility and cerebral perfusion (Kwater et al., 2009; Tarumi et al., 2011; Webb et al., 2012). Studies to date examining associations between arterial stiffness and cerebrovascular pulsatility have been cross-sectional. It is largely unknown if acute elevations in arterial stiffness affect cerebrovascular pulsatility.

Resistance exercise (RE) is an accepted lifestyle strategy to increase muscular strength and attenuate sarcopenia-related functional impairment (Miyachi et al., 2004; Miyachi, 2013) and is currently recommended by major health organizations for health promotion/disease prevention (Pollock et al., 2000). Paradoxically, despite functional and musculoskeletal benefits, RE may impair vascular function (Miyachi et al., 2004; Heffernan et al., 2007b; Collier et al., 2010). Higher intensity RE training typically increases arterial stiffness (Miyachi, 2013) and recent evidence suggests that RE training may contribute to reductions in cerebral blood flow velocity (CBFv) (Jung et al., 2012). Acute RE has consistently been shown to increase large central artery stiffness (Heffernan et al., 2007a; Collier et al., 2010) immediately after and for upwards of 30 min after perturbation (DeVan et al., 2005). Given the strong cross-sectional association between arterial stiffness, pressure/flow pulsatility, and cerebral perfusion, it is possible that acute RE-induced increases in arterial stiffness may transiently increase CBFv pulsatility. The purpose of this study was to test the hypothesis that acute RE-induced increases in arterial stiffness would lead to increases in carotid pressure/flow pulsatility and CBFv pulsatility.

## Methods

### Participants

Eighteen recreationally active men, ranging in age from 19–28 yrs, were recruited from the local University community for this randomized, crossover-design study. None were highly trained in an exclusive exercise modality (i.e., distance runners, bodybuilders, or power lifters). Exclusion criteria included self-reported smoking, hypertension, diabetes mellitus, hyperlipidemia, pulmonary disease, renal disease, neurological disease or peripheral artery disease and/or use of medications of any kind. This study was approved by the Institutional Review Board of Syracuse University and all participants provided written informed consent prior to study initiation. Vascular testing was conducted at the same time of day in a quiet, dimly lit, temperature-controlled laboratory. Participants were instructed to fast for ≥3 h and avoid vigorous exercise and consuming caffeine/alcohol ≥12 h before testing. Height and weight were assessed via wall-mounted ruler and electronic scale, respectively, and body composition was estimated via air displacement plethysmography (BodPod; COSMED, Concord, CA).

### Design

Upon arrival, participants rested for 10 min in the supine position. This was followed by all vascular and hemodynamic measures. Participants then performed (a) upper body RE, or (b) seated rest with minimal movement (time control) in a randomized order on two separate days. The RE protocol consisted of 5 sets of 5-repetitions for bench press and 5 sets of 10-repetitions for biceps curl, with each set being separated by 90 s of rest. Exercises were performed at 100% of participants 5 repetition maximum (RM) and 10 RM for bench press and biceps curl respectively. If participants could not complete the designated number of repetitions, a “drop set” was instituted whereby the total load was reduced in small increments until participants successfully completed target repetitions. Participants RMs for each exercise were determined previously on a separate day. This acute RE protocol has previously been shown to acutely increase arterial stiffness (Fahs et al., 2009). Post-testing vascular and hemodynamic measures were made 10, 20, and 30 min following acute RE based on previous findings noting prominent changes in arterial stiffness to occur at these times (DeVan et al., 2005; Heffernan et al., 2007a; Fahs et al., 2009). Carotid pressure, flow, stiffness, and cerebral velocity measures were obtained simultaneously.

### Measures

#### Brachial blood pressure

Systolic blood pressure (SBP) and diastolic brachial blood pressure (DBP) was measured prior to each set of vascular measures (baseline, post-1, post-2, post-3) using a validated, automated oscillometric cuff (EW3109, Panasonic Electric Works, Secaucus NJ). Pressures were taken in duplicate and averaged. If values were different by more than 5 mmHg a third measure was obtained and the average of the 2 closest measures used for subsequent analyses.

#### Carotid doppler ultrasonography

Images of the common carotid artery (CCA) were obtained using Doppler ultrasound (ProSound α7, Aloka, Tokyo, Japan) and 7.5–10.0 mHz linear-array probe. CCA intima-media thickness (IMT) was assessed using a longitudinal view with both near wall and far wall lumen-IMT boundaries visible. Wall thickness was measured across a 5 mm region of interest via semi-automated digital calipers during systole and diastole (determined from ECG gating). The distance from the lumen-intima interface to the media-adventitia interface was measured as the IMT. CCA systolic and diastolic diameters were measured from inside the near-wall IMT to far-wall IMT. CCA mean blood velocities (*Vm*) were measured using Doppler-ultrasound and calculated as: *Vm* = ∫*V*(*t*)*dt*/*FT*, where ∫*V*(*t*)*dt* is the velocity-time integral of the velocity waveform and FT is flow time. CCA flow and shear rate were calculated as π × (1/3 systolic radius + 2/3 diastolic radius)^2^ × *Vm* × 60 and 4 × (*Vm*/systolic diameter), respectively. CCA mean circumferential wall tension was calculated as [mean arterial pressure x mean radius], where mean arterial pressure (MAP) is expressed in dyne·cm^2^. CCA tensile wall stress was calculated by dividing circumferential wall tension by IMT where both mean radius and IMT are expressed in cm (Carallo et al., 1999) CCA pulsatility index (PI) was calculated with a semi-automated flow tracing software using the following equation: (*V*_*s*_–*V*_*d*_)/mean *V*, where *V*_*s*_ is the peak systolic velocity, *V*_*d*_ diastolic velocity and mean *V* the mean velocity. CCA flow resistance index (RI) was calculated as (*V*_*s*_–*V*_*d*_)/*V*_*s*_.

Wave intensity analysis (WIA) combined with eTracking was used to derive forward and reflected wave intensity as measures related to pulsatile cerebrovascular burden and arterial stiffness in the carotid artery. WIA was performed simultaneously with carotid applanation tonometry on the contra-lateral artery. The distance from the near wall to far wall lumen-intima interface is continuously traced using eTracking software, creating a distension waveform almost identical to pressure waveforms (Van Bortel et al., 2001; Niki et al., 2002). WIA distension waveforms were calibrated against carotid systolic and diastolic pressures obtained via applanation tonometry described below. Flow waveforms were measured using range gated color Doppler signals averaged along the Doppler beam. An insonation angle ≤60° was maintained for all measures and sample volume was manually adjusted to encompass the entire vessel. At least 8 carotid waveforms were averaged to gain a representative average waveform. Wave intensity was calculated using time derivatives of blood pressure (P) and velocity (U), where wave intensity = (*dP/dt* × *dU/dt*); the area under the *dP/dt* × *dU/dt* curve represents the energy transfer of the wave (Sugawara et al., 2009b). W_1_ represents a forward compression wave produced during early systole, accelerating flow and increasing pressure; 2 the negative area (NA) occurring immediately after W_1_ is a backward travelling compression wave due to reflected waves from the periphery that decelerate flow but increase pressure. NA measured in the CCA has been suggesting as a measure of cerebrovascular tone (Bleasdale et al., 2003).

Arterial stiffness measures included beta stiffness index (β), and Peterson's pressure-strain elastic modulus (Ep) and were calculated as:

$$\beta =\ln({\text{P}}_{\text{Max}}/{\text{P}}_{\text{Min}})/[({\text{D}}_{\text{Max}}-{\text{D}}_{\text{Min}})/{\text{D}}_{\text{Min}}]$$

$$\text{Ep}=({\text{P}}_{\text{Max}}-{\text{P}}_{\text{Min}})/[({\text{D}}_{\text{Max}}-{\text{D}}_{\text{Min}})/{\text{D}}_{\text{Min}}]$$

where P and D correspond to pressure and diameter respectively, and Max and Min refer to maximum (systolic) and minimum (diastolic) values during the cardiac cycle. CCA characteristic impedance (Zc) was calculated by re-arranging the Water-Hammer equation as *Zc* = (*PWVx*ρ)/*A*, where *ρ* is blood density (assumed constant 1.055 kg/cm^3^) and A is carotid area. A one-point pulse wave velocity (PWV) was derived from local wave speed (*c*) as (β*P/2*ρ)^1/2^ (Harada et al., 2002; Hanya, 2013).

#### Carotid and aortic blood pressure waveform analysis

Pressure waveforms were obtained simultaneously in the contralateral CCA from a 10 s epoch and measured in duplicate using applanation tonometry (SphygmoCor, AtCor Medical, Syndey, Australia). CCA pressure waveforms were calibrated to brachial MAP and DBP. Additionally, aortic pressure waveforms were derived from radial pressure waveforms measured in duplicate (10 s epochs) and a generalized transfer function. Pulse pressure (PP) was calculated as SBP minus DBP. Augmentation index was calculated as the difference between the early- and late systolic peaks of the pressure waveforms to the total PP expressed as a percentage (P2-P1/PP × 100) and standardized to a heart rate of 75 beats per min (AIx75).

Wave separation analyses (WSA) were performed in order to obtain complimentary data to WIA and to gain further insight into origins of pressure pulsatility following acute RE. Pressure waveforms were separated into forward (Pf) and backwards/reflected (Pb) components. This technique uses a modified average-flow waveform based on the original flow triangulation method of Westerhof et al. and has been described previously in detail (Westerhof et al., 2006). Additionally, aortic pulse wave velocity (PWV) was estimated using the time lag between aortic Pf and Pb (Qasem and Avolio, 2008). Carotid-femoral distance was estimated by multiplying body height by 0.29 which may reduce measurement bias due to body disproportion that can occur with the standard tape measure method (Filipovsky et al., 2010).

#### Cerebral blood flow velocity

Middle cerebral artery (MCA) blood velocity was assessed using a 2-mHz transcranial Doppler (TCD) ultrasound probe (DWL Doppler Box-X, Compumedics, Germany) applied to the temporal window. Mean CBFv and pulsatility index were measured at a depth of 50–65 mm (Jung et al., 2012; Xu et al., 2012). The envelope of the velocity spectrum and mean velocity were calculated by a standard algorithm implemented on the instrument with use of a fast Fourier transform. MCA PI was calculated via an automated waveform tracking function using the same equation described for CCA PI. Cerebrovascular resistance index (CVRi) was calculated as MAP/*V*_mean_.

### Statistical analyses

All data is reported as mean ± standard error of the mean and statistical significance was established *a priori* as *p* < 0.05. Normality of distribution for variables was assessed qualitatively using histograms and Q-Q plots as well as quantitatively using the Shapiro-Wilk test. Data that were not normally distributed were log transformed to meet assumptions for parametric analyses. An analysis of variance with repeated measures (2 conditions × 4 time points) was used to analyze main outcome variables. *Post-hoc t*-tests were used to investigate significant interactions.

## Results

**Figure 1:**
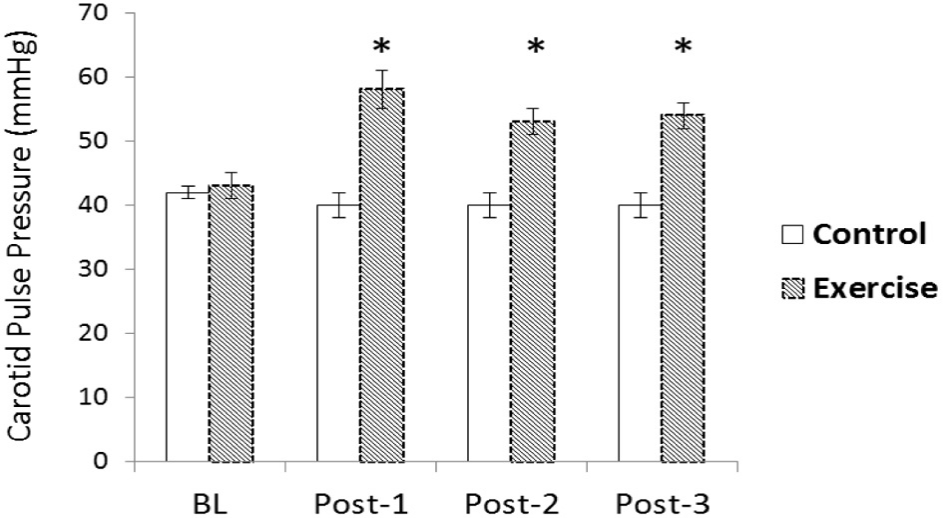
**Change in carotid pulse pressure (PP) following acute resistance exercise (RE) vs. a time control condition**. A significant condition-by-time interaction was detected for carotid PP (*p* < 0.05). Carotid PP was elevated 10, 20, and 30-min after exercise compared to baseline (*p* < 0.05)^*^.

Participants' age, body mass index, and body fat were, 22 ± 1 years, 23.7 ± 0.5 kg·m^−2^ and 11.4 ± 0.9%, respectively. The average load for the 5-RM bench press and 10-RM biceps curl were 76 ± 4 and 32 ± 2 kg, respectively. Carotid and cerebral measures are displayed independently in Tables 1–5. There were significant group-by-time interactions for brachial and carotid SBP, DBP, and PP. *Post-hoc* analyses revealed brachial SBP significantly increased immediately following RE and remained elevated for all post-RE time points compared to baseline (*p* < 0.05; Table 1). Similarly, carotid SBP at post-1 was significantly different from baseline following RE (*p* < 0.05; Table 1). Carotid SBP at post-2 and post-3 did not differ from baseline for the RE condition (*p* > 0.05). DBP decreased post-exercise compared to baseline for all time points resulting in overall increases in PP (*p* < 0.05; Table 1). There was a significant interaction detected for heart rate, as it was significantly elevated following RE for the duration of post-testing compared to baseline (*p* < 0.05; Table 1). Changes in carotid PP are displayed in Figure 1.

**Table 1:** **Common carotid artery (CCA) and brachial pressures across testing time points between control and resistance exercise (*n* = 18)**.

**Variable**	**Condition**	**Baseline**	**Post-1**	**Post-2**	**Post-3**	**Interaction**
Brachial SBP (mmHg)	Control	122 ± 1	121 ± 2	117 ± 2	119 ± 2	0.009
	RE	122 ± 1	131 ± 3^a^^,^^b^	126 ± 2	125 ± 2	
Brachial DBP (mmHg)	Control	74 ± 1	73 ± 1	71 ± 1^a^	73 ± 1	0.002
	RE	73 ± 1	65 ± 1^a^^,^^b^	65 ± 2^a^^,^^b^	66 ± 1^a^^,^^b^	
Brachial MAP (mmHg)	Control	90 ± 1	89 ± 1	86 ± 1	88 ± 1	0.220
	RE	89 ± 1	87 ± 1	85 ± 1	85 ± 1	
Brachial PP (mmHg)	Control	48 ± 1	48 ± 2	46 ± 2	46 ± 2	<0.001
	RE	49 ± 2	66 ± 3^a^^,^^b^	61 ± 2^a^^,^^b^	59 ± 2^a^^,^^b^	
CCA SBP (mmHg)	Control	115 ± 2	114 ± 2	110 ± 2	111 ± 2	0.043
	RE	116 ± 2	123 ± 3^a^^,^^b^	119 ± 3	120 ± 3	
CCA DBP (mmHg)	Control	73 ± 1	73 ± 1	70 ± 1^a^	72 ± 1	<0.001
	RE	73 ± 1	65 ± 1^a^^,^^b^	66 ± 2^a^^,^^b^	66 ± 1^a^^,^^b^	
CCA PP (mmHg)	Control	42 ± 2	40 ± 2	40 ± 2	40 ± 2	<0.001
	RE	43 ± 2	58 ± 4^a^^,^^b^	53 ± 3^a^^,^^b^	54 ± 3^a^^,^^b^	
Heart rate (b·min^−1^)	Control	60 ± 2	58 ± 2^a^	60 ± 3	58 ± 2^a^	<0.001
	RE	64 ± 3	86 ± 4^a^^,^^b^	82 ± 4^a^^,^^b^	79 ± 3^a^^,^^b^	

a *Significantly different from within-condition baseline, p < 0.05*.

b *Significantly different from other condition, same time point, p < 0.05*.

**Table 2:** **Common carotid dimensions/hemodynamics across testing time points between control and resistance exercise (*n* = 18)**.

**Variable**	**Condition**	**Baseline**	**Post-1**	**Post-2**	**Post-3**	**Interaction**
Mean diameter (mm)	Control	5.54 ± 0.09	5.55 ± 0.11	5.57 ± 0.10	5.69 ± 0.10	0.539
	RE	5.48 ± 0.11	5.35 ± 0.11	5.51 ± 0.11	5.54 ± 0.09	
Blood flow (mL·s^−1^)	Control	612.5 ± 21.3	656.6 ± 36.4	616.7 ± 20.8	626.3 ± 29.7	0.255
	RE	635.0 ± 27.6	664.8 ± 35.4	700.3 ± 28.8	672.5 ± 26.7	
Shear rate (sec^−1^)	Control	279.1 ± 13.1	293.7 ± 17.5	275.9 ± 14.2	259.3 ± 9.6	0.105
	RE	296.9 ± 12.7	339.9 ± 16.7	336.2 ± 15.9	305.76 ± 10.9	
Mean circumferential wall tension (dynes·cm^−1^)	Control	3.4 ± 0.1	3.4 ± 0.1	3.3 ± 0.1	3.4 ± 0.1	0.287
	RE	3.3 ± 0.1	3.2 ± 0.1	3.2 ± 0.1	3.2 ± 0.1	
Mean tensile stress (10^4^ dynes·cm^−1^)	Control	89.5 ± 2.9	92.2 ± 2.7	84.0 ± 2.9	91.4 ± 3.8	0.157
	RE	87.5 ± 3.2	81.2 ± 3.8	78.9 ± 3.1	81.5 ± 3.2	
Resistance Index	Control	0.77 ± 0.01	0.77 ± 0.01	0.76 ± 0.01	0.76 ± 0.01	0.013
	RE	0.76 ± 0.01	0.80 ± 0.01^a^^,^^b^	0.78 ± 0.01	0.78 ± 0.01	
Pulsatility Index	Control	2.07 ± 0.08	2.10 ± 0.08	2.06 ± 0.10	2.11 ± 0.09	0.094
	RE	2.01 ± 0.09	2.16 ± 0.08	1.95 ± 0.05	2.02 ± 0.06	

a *Significantly different from within-condition Baseline, p < 0.05*.

b *Significantly different from other condition, same time point, p < 0.05*.

**Table 3:** **Measures of common carotid wave reflection/stiffness across testing time points between control and resistance exercise (*n* = 18)**.

**Variable**	**Condition**	**Baseline**	**Post-1**	**Post-2**	**Post-3**	**Interaction**
β stiffness (AU)	Control	3.94 ± 0.37	3.63 ± 0.27	3.80 ± 0.24	3.53 ± 0.21	0.025
	RE	3.86 ± 0.35	5.30 ± 0.35^a^^,^^b^	4.82 ± 0.38^a^^,^^b^	5.00 ± 0.33^a^^,^^b^	
Ep (kPa)	Control	48.72 ± 4.76	44.28 ± 3.24	44.89 ± 3.03	42.06 ± 2.28	0.032
	RE	47.22 ± 4.11	64.78 ± 4.61^a^^,^^b^	56.33 ± 4.26^a^^,^^b^	59.78 ± 4.15^a^^,^^b^	
Zc (DSC)	Control	1846 ± 103	1796 ± 90	1777 ± 70	1649 ± 45	0.094
	RE	1868 ± 89	2215 ± 100	1959 ± 87	1977 ± 68	
W_1_ (mmHg·m·sec^−3^)	Control	9.39 ± 0.84	10.65 ± 1.25	9.66 ± 1.24	9.13 ± 0.80	0.015
	RE	9.31 ± 0.97	16.61 ± 2.04^a^^,^^b^	13.51 ± 1.45^a^^,^^b^	11.71 ± 1.31^a^	
lnNA (mmHg·m·s^−2^)	Control	3.25 ± 0.45	3.58 ± 0.30	3.47 ± 0.23	3.27 ± 0.28	0.160
	RE	3.85 ± 0.35	4.89 ± 0.47	4.22 ± 0.42	3.22 ± 0.47	
AIx75 (%)	Control	−25 ± 2	−26 ± 3	−27 ± 3	−27 ± 3	0.945
	RE	−29 ± 3	−30 ± 3	−31 ± 2	−31 ± 3	
**WAVE SEPARATION (*n* = 12)**						
Pf (mmHg)	Control	40 ± 2	38 ± 3	44 ± 5	39 ± 2	<0.001
	RE	41 ± 3	58 ± 4^a^^,^^b^	47 ± 3^a^^,^^b^	49 ± 4^a^^,^^b^	
Pb (mmHg)	Control	16 ± 1	15 ± 1	15 ± 1	16 ± 1	0.649
	RE	15 ± 1	16 ± 1	14 ± 1	16 ± 1	

a *Significantly different from within-condition BL, p < 0.05*.

b *Significantly different from other condition, same time point, p < 0.05*.

*AIx75, augmentation index at a heart rate of 75 bpm; NA, negative area; Pf, forward pressure; Pb, backwards pressure, DSC, dynes x s/cm^5^*.

**Table 4:** **Measures of aortic wave reflection/stiffness across testing time points between control and resistance exercise (*n* = 12)**.

**Variable**	**Condition**	**Baseline**	**Post-1**	**Post-2**	**Post-3**	**Interaction**
PWV (m·s^−1^)	Control	7.4 ± 0.2	7.4 ± 0.2	7.3 ± 0.2	7.1 ± 0.2	0.080
	RE	7.1 ± 0.1	8.1 ± 0.2	7.6 ± 0.1	7.3 ± 0.1	
Pf (mmHg)	Control	32 ± 1	34 ± 2	34 ± 2	36 ± 3	0.867
	RE	35 ± 2	35 ± 3	38 ± 3	40 ± 2	
Pb (mmHg)	Control	13 ± 1	14 ± 1	14 ± 1	13 ± 1	0.249
	RE	12 ± 1	15 ± 1	13 ± 1	13 ± 1	
Time to Pb (ms)	Control	284 ± 14	276 ± 11	278 ± 15	286 ± 15	0.040
	RE	272 ± 8	249 ± 5^a^^,^^b^	246 ± 4^a^^,^^b^	246 ± 5^a^^,^^b^	
AIx75	Control	−11 ± 3	−8 ± 4	−13 ± 4	−11 ± 3	0.013
	RE	−9 ± 2	7 ± 4	1 ± 3^a^^,^^b^	−3 ± 3^a^^,^^b^	

a *significantly different from within-condition BL, p < 0.05*.

b *significantly different from other condition, same time point, p < 0.05*.

*AIx75, augmentation index at a heart rate of 75 bpm; PWV, pulse wave velocity; Pf, forward pressure; Pb, backwards pressure*.

**Table 5:** **Cerebral variables across testing time points between control and resistance exercise (*n* = 18)**.

**Variable**	**Condition**	**Baseline**	**Post-1**	**Post-2**	**Post-3**	**Interaction**
Mean velocity (cm·s^−1^)	Control	56 ± 5	56 ± 4	56 ± 4	56 ± 5	0.491
	RE	57 ± 4	54 ± 4	55 ± 4	55 ± 4	
Pulsatility index	Control	0.85 ± 0.03	0.85 ± 0.04	0.86 ± 0.03	0.84 ± 0.04	0.325
	RE	0.87 ± 0.03	0.89 ± 0.03	0.84 ± 0.03	0.83 ± 0.03	
Resistance index (mmHg^−1^·cm·s^−1^)	Control	1.79 ± 0.13	1.71 ± 0.12	1.68 ± 0.12	1.74 ± 0.12	0.162
	RE	1.67 ± 0.11	1.76 ± 0.13	1.65 ± 0.10	1.72 ± 0.13	

**Figure 2:**
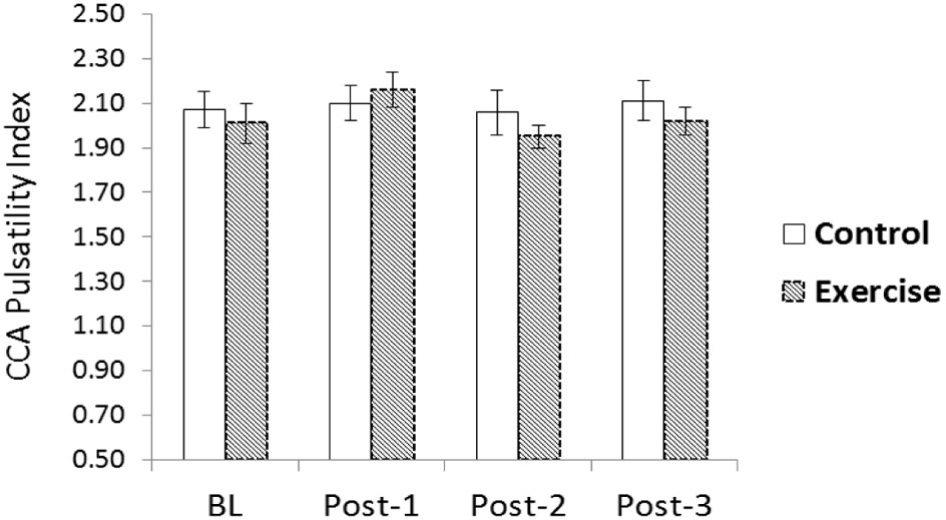
**Common carotid artery (CCA) blood flow velocity pulsatility index (PI) following acute resistance exercise (RE) vs. a time control condition**. No significant changes were noted (*p* > 0.05).

**Figure 3:**
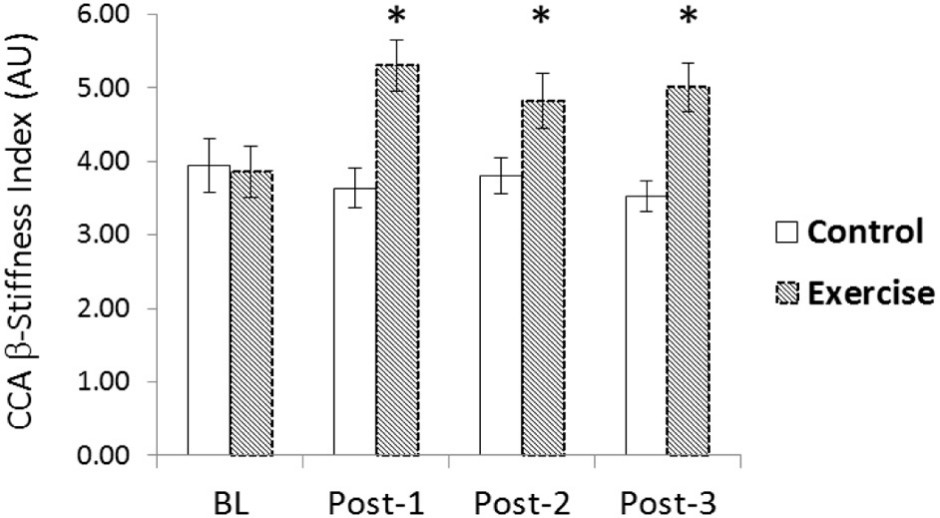
**Change in common carotid artery (CCA) β-stiffness index following acute resistance exercise (RE) vs. a time control condition**. A significant condition-by-time interaction was detected for (CCA) β-stiffness index (*p* < 0.05). β-stiffness index was elevated 10, 20, and 30-min after exercise compared to baseline (*p* < 0.05)^*^.

There were no significant changes in CCA systolic or diastolic IMT (data not shown), CCA mean blood flow, or CCA PI following acute RE (Table 2 and Figure 2). Both CCA Ep and β-stiffness significantly increased following RE, and remained significantly elevated throughout post-testing (*p* < 0.05; Table 3). Changes in β-stiffness are displayed in Figure 3. A condition-by-time interaction was detected for carotid W_1_, which significantly increased post-RE and remained elevated throughout post-testing (*p* < 0.05; Table 3). Carotid AIx75 was not significantly altered by acute RE (*p* > 0.05). NA was not normally distributed and was log transformed to meet assumptions for parametric statistical analyses. There were no statistically significant changes in carotid lnNA (*p* > 0.05). WSA could not be performed for 6 participants as reflection time was outside of the measurement device's acceptable range (<50 ms) for calculations. Data for the remaining 12 are reported herein. Carotid Pf had a significant condition-by-time interaction; *post-hoc* testing revealed Pf significantly increased following RE, remaining elevated through post-3 (*p* < 0.05; Table 3). There were no statistically significant changes in carotid Pb (*p* > 0.05).

**Figure 4:**
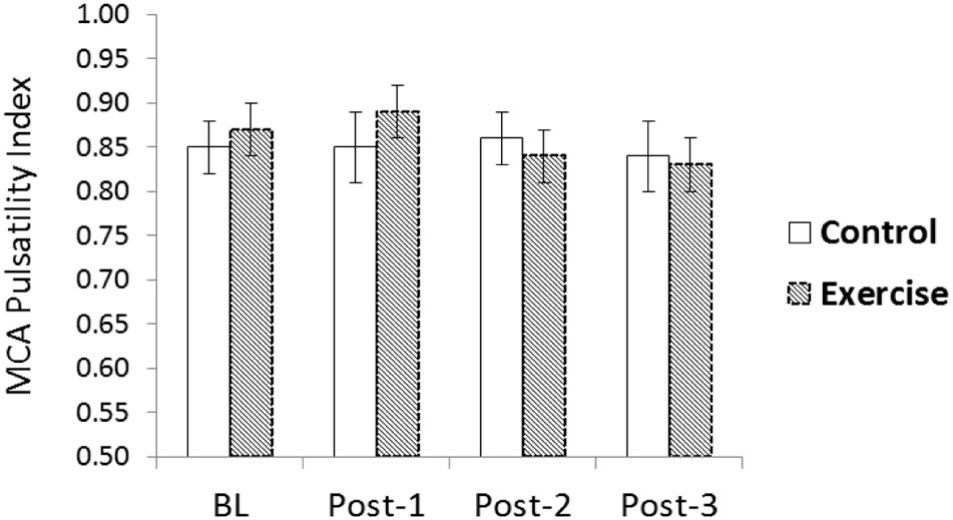
**Middle cerebral artery (MCA) blood flow velocity pulsatility index (PI) following acute resistance exercise (RE) vs. a time control condition**. No significant changes were noted (*p* > 0.05).

There was a significant interaction for aortic AIx75 (*p* < 0.05) and a trend for an interaction for aortic stiffness as values were elevated following acute RE but not following the time control (*p* = 0.08; Table 4). There was no significant change in aortic Pf or Pb following acute RE (*p* > 0.05). A significant condition-by-time interaction was detected for aortic time to Pb, indicative of faster wave reflection speed. Values significantly decreased post-RE compared to baseline and remained different through post-3 (*p* < 0.05). Mean MCA CBFv, MCA PI (Figure 4), and MCA CVRi did not differ across time or between conditions (*p* > 0.05, Table 5).

## Discussion

The novel finding of the present study was that while acute RE does increase carotid artery stiffness and pressure pulsatility, acute RE may not affect carotid or cerebral flow pulsatility. Moreover, using novel methods of hemodynamic appraisal (WIA and WSA) we provide unique insight into the origin of increased pressure pulsatility after acute RE; increased pressure pulsatility in the carotid artery is likely due to increased forward wave pressure (Pf and W_1_) and not pressure from wave reflections (Pb or NA).

Studies examining cerebrovascular responses to RE have traditionally focused on the steady component of pressure-flow relationships during and immediately after (<60 s) RE and whether changes in mean arterial pressure alter cerebral autoregulation (Edwards et al., 2002; Pott et al., 2003; Koch et al., 2005). The current study was designed to investigate changes in *pulsatile* hemodynamics (not the steady component or mean flow) during previously established times of elevated arterial stiffness following RE. The steady and pulsatile components of pressure/flow each differentially associate with cerebral structure and function (Mitchell et al., 2011). Many studies note no associations between mean cerebrovascular inflow and cerebral structure/function (Bateman, 2002; Patankar et al., 2006; Henry-Feugeas et al., 2009; Jolly et al., 2013). Conversely, pulsatile flow has been shown to more consistently associate with cerebrovascular damage (Mitchell et al., 2011; Webb et al., 2012; Jolly et al., 2013).

In the present study, despite substantial increases in carotid stiffness for upwards of 30-min after acute RE, there were minimal change in CBFv pulsatility. Carotid artery stiffness increased following RE, reinforcing previous observations regarding the acute effects of RE on central artery stiffness (DeVan et al., 2005; Heffernan et al., 2007b; Fahs et al., 2009; Collier et al., 2010; Yoon et al., 2010). Increased arterial stiffness has previously been linked to pulsatile *flow* in the cerebrovascular bed (Xu et al., 2012), higher white matter hyperintensities, lower executive function, and increased risk for subcortical infarcts (Mitchell, 2008) separate from the effects of pressure pulsatility. In the current study, RE-induced increases in arterial stiffness did not alter measures of CCA or MCA flow pulsatility. Thus physiological changes in arterial stiffness measured 10–30 min after acute RE may not alter cerebral flow profiles as occurs inherently/chronically with aging or in pathological settings.

Arterial stiffness has also been associated with pulsatile *pressure* (Tarumi et al., 2011) and in turn impaired cerebrovascular function (Kwater et al., 2009; Webb et al., 2012). In the present study there were increases in carotid pressure pulsatility evidenced by a significant increase in carotid PP post-RE. The blood pressure waveform is an amalgam of forward and backward travelling waves. Left ventricular ejection instigates the genesis of forward travelling pressure waves. These pressure waves may be partially reflected from peripheral vessels with the timing and magnitude of this reflection affected by several hemodynamic factors including arterial stiffness, peripheral vascular tone, and physical distance to the peripheral reflection sites. Using two different novel yet complementary methods of assessing carotid hemodynamics (WIA and WSA), the present study noted that increased pressure pulsatility in the CCA was largely driven by an increase in forward wave pressure (increases in Pf and W_1_) as there were no statistically significant changes in pressure from wave reflections (Pb or NA). This is consistent with recent findings from Schultz et al. noting that increases in central pressure during exercise are largely mediated by increases in forward wave pressure and not pressure from wave reflections (Schultz et al., 2013). It should be underscored that changes in central hemodynamics following acute RE may not be completely devoid of risk. Clinical consequences of increased forward wave pressure and subsequent pulsatile pressure transmission to the cerebrovascular bed following acute RE require further scrutiny.

Wave reflections detected in the CCA have been shown to be related to altered cerebrovascular tone (Bleasdale et al., 2003; Curtis et al., 2007). Increases in cerebral resistance affect the timing and magnitude of pressure waves being reflected from the head (cerebral circulation) (Bleasdale et al., 2003; Curtis et al., 2007). In general, resistance in the cereobrovascular bed is low allowing for possible transmission of damaging pulsatile energy to penetrate deeply into the intracranial cavity. An increase in cerebrovascular tone would increase pressure from wave reflections (from the brain to the carotid) and might serve to protect against entry of pulsatile energy into the delicate cerebral capillary bed. An increase in wave reflections from the cerebral vasculature would be expected to increase carotid pressure but attenuate flow (Kohara et al., 1999; Curtis et al., 2007). In the current study, there were no statistically significant changes in carotid NA or Pb (measures of wave reflections) and this mirrored the lack of change seen in carotid and cerebral flow measures. Thus despite large increases in forward wave *pressure* and overall *pressure* pulsatility, RE and/or RE-induced stiffness may not alter cerebrovascular *flow* pulsatility at the time points investigated. In young healthy adults, cerebrovascular flow pulsatility may be dampened via other mechanisms.

Our data offer insight into flow pulsatility buffering following acute RE and suggest that some may occur at the level of the CCA. It has been suggested that as much as a quarter to half of cerebrovascular resistance may be determined at the level of the carotid arteries (Willie et al., 2014). In the present study, the modest increases in CCA Zc after acute RE were associated with reduced CCA PI (*r* = −0.61, *p* < 0.05). Eighty percent of CCA blood flow at rest feeds the internal carotid artery with subsequent branching giving way to the MCA that in turn supplies approximately 80% of the blood supply to the brain (Farkas and Luiten, 2001). Compared to the external carotid artery (ECA), the internal carotid artery (ICA) is a lower resistance/impedance vessel with a lower reflection coefficient (Taylor and Tukmachi, 1985) favoring a flow differential at rest. The input impedance of the CCA is thus largely determined by the ECA; parenthetically, the CCA flow pattern approximates that of the lower impedance ICA rather than the ECA (Taylor and Tukmachi, 1985). Owing to the intracranial anastomoses through the orbit, ICA occlusion results in flow redistribution to the ipsilateral ECA and a change in the ECA flow profile to match that of a typical ICA/CCA contour (Taylor and Tukmachi, 1985). During exercise, it has been shown that there is an increase in ECA flow relative to the ICA for thermoregulatory purposes (Sato et al., 2011). Taken together and these observations suggest that changes in CCA/ICA/ECA impedance matching during/following exercise may affect pulsatile flow transmission at these crucial interfaces.

Some buffering of hemodynamic pulsatility may also occur at the carotid-aortic interface (Mitchell et al., 2011). We noted disparate changes in carotid and aortic hemodynamics following acute RE. While there was no change in carotid AIx, there was a significant increase in aortic AIx following acute RE (Yoon et al., 2010). Increases in aortic AIx were due to changes in timing of wave reflection travel likely from an increase in aortic PWV and not changes in magnitude as there were minimal changes in aortic Pb. Arterial stiffness and pressure from wave reflections have been shown to affect retrograde flow in the femoral/aorta (Hashimoto and Ito, 2010; Heffernan et al., 2013) which has been linked to flow in the CCA (Hashimoto and Ito, 2013). There were also disparate changes in forward wave pressure (Pf) between CCA and aorta. Thus, alterations in impedance matching at the carotid-aorta interface may also affect CCA pressure and flow pulsaility following acute RE. An additional alternative mechanism for buffering of flow pulsatility to the MCA may reside in the geometry of the ICA. The carotid siphon describes the tortuous distal part of the internal carotid artery that may bend beyond 180° with minimal inter-individual variability (Schubert et al., 2011). This shape has been shown to enhance pulsatile energy dispersion and markedly attenuate flow pulsatility *in vivo* (Schubert et al., 2011). Finally, it is also possible that some hemodynamic buffering occurs within the intracranial vessels via their capacitance properties. The cerebral Windkessel may extinguish pulsatilite hemodynamic energy via matching cerebrospinal fluid ejection to venous flow volume out of the intracranial cavity (Chan et al., 2011).

Elevations in HR after acute RE support previous studies noting increases in sympathetic nervous system (SNS) activity during recovery from this exercise modality (Heffernan et al., 2006). The role of the SNS in modulating cerebral hemodynamics in humans remains conflicting (Willie et al., 2014). While select studies suggest an important role for the SNS in affecting mean flow patterns and autoregulation in cerebral vessels, studies directly examining associations between the SNS and cerebrovascular flow pulsatility are lacking. The effect of SNS activity on carotid artery mechanical properties in humans are also conflicting with some studies suggesting a link (Sugawara et al., 2009a; Liu et al., 2011) and others noting no association (Kosch et al., 2002). It is possible that increases in sympathetic activation following acute RE contribute to increases in CCA stiffness/impedance while concomitantly preventing forced dilation of the cerebral arterioles; both of which may prevent regional over-perfusion and protect against blood-brain barrier breakdown from transmission of excessive pressure/flow fluctuations (Ogoh and Ainslie, 2009).

MCA PI is elevated immediately (within 2-min) after acute RE (Koch et al., 2005) thus it is possible an important window for data acquisition was missed. We intentionally chose not to assess vascular properties during this time point as marked changes in MAP, HR and ventilation with subsequent changes in CO_2_ could confound interpretation of findings (Wilkinson et al., 2002; Romero and Cooke, 2007). Of central importance for the present study and in accordance with previously published studies (DeVan et al., 2005) CCA stiffness was elevated at all time-points assessed. Despite these changes in CCA stiffness, there was no change in CCA or MCA flow pulsatility at these time points reinforcing our conclusion that acute RE-mediated increases in arterial stiffness may not detrimentally impact cerebrovascular flow pulsatility. Future research is needed to explore central hemodynamic and cerebrovascular changes during earlier time points following acute RE.

### Limitations

Additional limitations to this study should be noted. This study utilized healthy young men. Results may not be directly applicable to women, older adults or other clinical populations. TCD cannot measure blood flow *per se* since diameter is not measured. Although previous research has suggested that the diameter of large cerebral vessels do not change across different physiological stimuli (Stroobant and Vingerhoets, 2000), this has never been demonstrated empirically following acute exercise. The WSA method used in this study was not completed on 6 participants due to wave reflection times occurring outside of the analysis software's acceptable range (reflection times <50 ms). This occurred most notably after acute RE. It is possible that RE-induced tachycardia and the concomitant decrease in ejection duration changed wave reflection timing such that reflected waves arrived during early systole. Despite incomplete results using this technique, values obtained from the subset of participants with complete CCA WSA data confirmed findings from CCA WIA. Finally, it must be noted that cerebral autoregulation was not measured as it was not the purpose of this investigation. Cerebrovascular regulation is preserved following acute aerobic exercise (Willie et al., 2013). Future research is needed to explore associations between CCA stiffness and cerebral autoregulation during/following various physiologic stressors including resistance exercise.

### Conclusion

Acute RE increases carotid artery stiffness and pressure pulsatility without affecting cerebral flow pulsatility. RE-induced increases in carotid pressure pulsatility may be due to increases in forward wave pressure and not pressure from wave reflections.

### Conflict of interest statement

The authors declare that the research was conducted in the absence of any commercial or financial relationships that could be construed as a potential conflict of interest.

## Abbreviations

RE: Resistance exercise
CBFv: Cerebral blood flow velocity
RM: Repetition maximum
SBP: Systolic blood pressure
DBP: Diastolic blood pressure
CCA: Common carotid artery
IMT: Intima-media thickness
MAP: Mean arterial pressure
PI: Pulsatility index
WIA: Wave intensity analysis
NA: Negative area
Ep: Elastic modulus
β: β Stiffness index
PP: Pulse pressure
Aix: Augmentation index
WSA: Wave separation analysis
Pf: Forward wave pressure
Pb: Backwards wave pressure
MCA: Middle cerebral artery
TCD: Transcranial Doppler
CVRi: Cerebrovascular resistance index.
